# TGF-β-Mediated Sustained ERK1/2 Activity Promotes the Inhibition of Intracellular Growth of *Mycobacterium avium* in Epithelioid Cells Surrogates

**DOI:** 10.1371/journal.pone.0021465

**Published:** 2011-06-22

**Authors:** Carolina L'Abbate, Ivone Cipriano, Elizabeth Cristina Pérez-Hurtado, Sylvia Cardoso Leão, Célia Regina Whitaker Carneiro, Joel Machado

**Affiliations:** 1 Disciplina de Imunologia, Departamento de Microbiologia, Imunologia e Parasitologia, Universidade Federal de São Paulo, São Paulo, Brasil; 2 Disciplina de Biologia do Desenvolvimento, Departamento de Morfologia e Genética, Universidade Federal de São Paulo, São Paulo, Brasil; 3 Disciplina de Microbiologia, Departamento de Microbiologia, Imunologia e Parasitologia, Universidade Federal de São Paulo, São Paulo, Brasil; 4 Departamento de Ciências Biológicas, Campus de Diadema, Universidade Federal de São Paulo, São Paulo, Brasil; Emory Unviersity, United States of America

## Abstract

Transforming growth factor beta (TGF-β) has been implicated in the pathogenesis of several diseases including infection with intracellular pathogens such as the *Mycobacterium avium* complex. Infection of macrophages with *M. avium* induces TGF-β production and neutralization of this cytokine has been associated with decreased intracellular bacterial growth. We have previously demonstrated that epithelioid cell surrogates (ECs) derived from primary murine peritoneal macrophages through a process of differentiation induced by IL-4 overlap several features of epithelioid cells found in granulomas. In contrast to undifferentiated macrophages, ECs produce larger amounts of TGF-β and inhibit the intracellular growth of *M. avium*. Here we asked whether the levels of TGF-β produced by ECs are sufficient to induce a self-sustaining autocrine TGF-β signaling controlling mycobacterial replication in infected-cells. We showed that while exogenous addition of increased concentration of TGF-β to infected-macrophages counteracted *M. avium* replication, pharmacological blockage of TGF-β receptor kinase activity with SB-431542 augmented bacterial load in infected-ECs. Moreover, the levels of TGF-β produced by ECs correlated with high and sustained levels of ERK1/2 activity. Inhibition of ERK1/2 activity with U0126 increased *M. avium* replication in infected-cells, suggesting that modulation of intracellular bacterial growth is dependent on the activation of ERK1/2. Interestingly, blockage of TGF-β receptor kinase activity with SB-431542 in infected-ECs inhibited ERK1/2 activity, enhanced intracellular *M. avium* burden and these effects were followed by a severe decrease in TGF-β production. In summary, our findings indicate that the amplitude of TGF-β signaling coordinates the strength and duration of ERK1/2 activity that is determinant for the control of intracellular mycobacterial growth.

## Introduction

Bacteria of the *Mycobacterium avium* complex are facultative intracellular microorganisms that mainly infect mononuclear phagocytes and cause disseminated infection in immunocompromised patients [1], [2]. The mycobacteria gain access to host tissues by a variety of portals [3], [4], and invade the mucosa via epithelial cells [5], [6]. Once the bacteria are transported into the deeper tissues by macrophages and perhaps other phagocytic cells, additional macrophages gather at individual infected foci to form granulomas. Granulomas likely begin as aggregates of mononuclear phagocytes that surround individual infected macrophages [7], [8]. These macrophages become activated, a transformation reflected by an increase in their size and subcellular organelles, ruffled cell membranes, and enhanced phagocytic and microbicidal capabilities [9], [10]. A common feature of all *Mycobacterium* granulomas is the differentiation of macrophages into epithelioid cells that have tightly interdigitated cell membranes in zipper-like arrays linking adjacent cells [7], [10]. It is speculated that mycobacteria enter into a latent phase within mature granulomas and remain contained within these structures for prolonged periods of time [11].

Several studies have shown that the transforming growth factor β (TGF-β) is produced by macrophages upon infection with *Mycobacterium* and also with other pathogens and that the presence of TGF-β favors virulence, probably through its immunosuppressive action by impairing the response of macrophages to cytokines such as TNF-α or by suppression of nitric oxide (NO), and reactive oxygen intermediates [12], [13], [14]. TGF-β is a pleiotropic cytokine produced by every leukocyte lineage, including lymphocytes, macrophages, and dendritic cells, and its expression serves in both autocrine and paracrine modes to control the differentiation, proliferation, and state of activation of these immune cells [15], [16]. The action of TGF-β on these cells is dependent not only on the cell type and its state of differentiation, but also on the milieu of cytokines present [17], suggesting that perturbations of the balance of this cytokine array may also alter effects of TGF-β and contribute to immunopathology. TGF-β exerts its effect through heteromeric receptor complex consisting of type I and type II transmembrane serine/threonine kinase receptors. Upon ligand binding TGF-β receptor recruits and activates Smad family members or activates Smad-independent signaling pathways such as the mitogen-activated protein kinase (MAPK) pathway [18]. Although TGF-β regulates several MAPK pathways, the mode of activation appears to be highly variable and cell type-dependent [18], [19], [20].

Besides the role of TGF-β favoring mycobacterium replication, it has been shown that mycobacterium can directly interfere with host-cell signaling pathways. Examples are the studies showing that entry of a virulent *M. avium* strain causes little activation of the p38 and ERK1/2 MAPKs in macrophages, whereas low or intermediated strain are potent inducers [21], [22], [23]. Moreover, inhibition of the ERK1/2 but not of the p38 pathway further enhanced intracellular growth of highly replicative *M. avium* strains, showing that pathogenic mycobacteria have evolved mechanisms to prevent a sustained activation of ERK1/2, and this may account for their intracellular growth [21]. Despite significant advances concerning the mechanisms of interaction and the consequent changes that are induced by mycobacterium in the macrophage signaling machinery, critical gaps remain in our understanding about a possible relationship between TGF-β signaling and ERK1/2 controlling mycobacterium replication in infected cells.

We have previously shown that epithelioid cell surrogates (ECs) derived from primary murine peritoneal macrophages through a process of differentiation induced by recombinant IL-4 (rIL-4), overlap several morphological and functional features of epithelioid cells found in granulomas [24]. In contrast to undifferentiated macrophages, ECs secrete high amounts of TGF-β and are efficient in inhibiting the intracellular growth of *M. avium* [25]. In the present study, we employed this cell system to investigate whether there is a relationship between the magnitude of TGF-β signaling and activation of ERK1/2 in the regulation of intracellular growth kinetics of *M. avium*.

## Results

### The type II IL-4R is the functional receptor involved in the signaling pathway leading to macrophage differentiation into ECs

**Figure 1:**
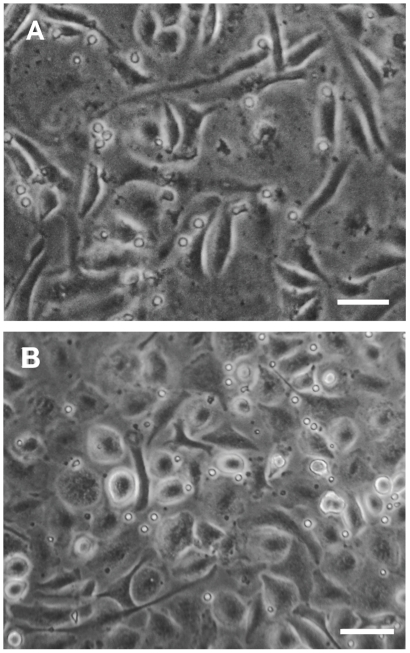
Primary peritoneal macrophages treated with rIL-13 display morphological features of ECs. (A) Untreated primary peritoneal macrophages and (B) primary peritoneal macrophages pulsed with rIL-13 (10 ng ml^−1^) on day one. After 7 days, cell monolayers were examined for morphological changes by phase contrast inverted light microscopy. Scale bar = 50 µm.

**Figure 2:**
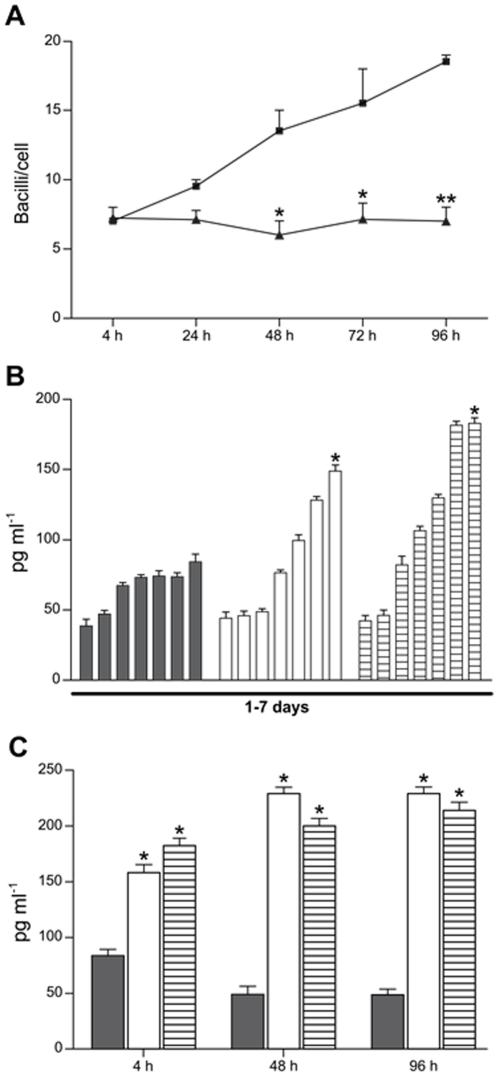
rIL-13-treated macrophages inhibit *M. avium* replication and secrete high amounts of TGF-β. (A) Kinetics of *M. avium* infection expressed as bacilli/cell in control macrophages (squares) and in rIL-13-treated macrophages (triangles) cultures. Results are means ± S.E.M. from a representative experiment among two carried out in duplicates. *P<0.05 and **P<0.01 when untreated macrophages are compared to rIL-13 treated macrophages at the same time point. (B) TGF-β measurement expressed as mean concentration ± S.E.M. obtained day by day from culture supernatants of macrophages pulsed either with rIL-4 (open bars) or rIL-13 (hatched bars), by sandwich ELISA. Black bars represent the mean concentration of TGF-β in untreated macrophages cultures. (C) TGF-β measurement in the cell culture supernatant of *M. avium*-infected cells. Infected-macrophages (black bars), infected-ECs (open bars) and infected rIL-13-treated macrophages (hatched bars). *P<0.05 when ECs and rIL-13-treated cells were compared to control macrophages at the same time point.

We have previously described that primary murine peritoneal macrophages pulsed with recombinant IL-4 (rIL-4) acquire morphological and functional characteristics of epithelioid cells after 7 days in culture [24], suggesting that signaling through IL-4 receptor is involved in the differentiation of macrophages into epithelioid cells surrogates (ECs). IL-4 is structurally similar and shares a functional signaling receptor chain with IL-13 [26], [27] and therefore, both cytokines can hold similar biological and immunoregulatory functions. Based on this premise we asked whether IL-13 could also promote differentiation of macrophages into ECs. To test this, peritoneal macrophages were pulsed with recombinant IL-13 (rIL-13) and after 7 days morphological and functional analysis were performed. As shown in Fig. 1, in contrast to untreated macrophages which showed morphology elongated, rIL-13-treated cells became grouped together and assumed a “fried-egg” shape, similar to ECs as previously described by Cipriano *et al* [24]. Among the functional features of ECs those include the ability to overcome intracellular *M. avium* replication as well as overproduction of TGF-β [25]. As shown in Fig. 2A, the behavior of rIL-13-treated macrophages upon infection with *M. avium* revealed that the number of bacilli/cell was lower throughout the course of infection, compared to the increased growth seen in untreated cells. Also the kinetics of TGF-β production during the 7 days culture of macrophages pulsed on day one with rIL-13 or rIL-4 were similarly increased in a time dependent manner (Fig. 2B). Moreover, TGF-β production was also as much higher in rIL-13-treated macrophages as in ECs during the course of *M. avium* infection (Fig. 2C). Taking together these results show that like rIL-4, rIL-13 was capable to induce morphological and functional changes that are representative of ECs, suggesting that both cytokines induce the same functional receptor involved in the differentiation process.

### High TGF-β levels produced by ECs mediate the inhibition of *M. avium* replication in infected-cells

**Figure 3:**
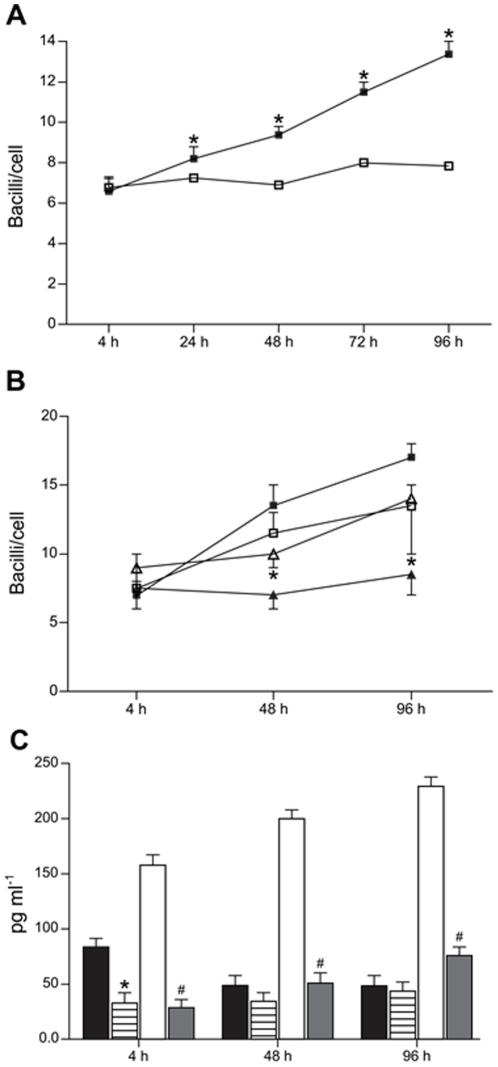
TGF-β signaling is involved in the regulation of intracellular *M. avium* growth. (A) Addition of rTGF-β into *M. avium*-infected macrophages. Results show the number of bacilli/cell in macrophages treated with rTGF-β (100 pg ml^−1^) 4 h post-infection, followed by rTGF-β addition each 24 h during the course of infection. *P<0.05 when treated macrophages (open squares) where compared to untreated macrophages (full squares) at each time point. Results are representative of two independent experiments done in duplicates. (B) Number of bacilli/cell in *M. avium*-infected cells. (Open triangles) SB431542-treated ECs, (open squares) SB-431542-treated macrophages, (full triangles) untreated ECs and (full squares) untreated macrophages. SB431542 (100 µM) was added 1 h before cell/bacilli co-incubation. *P<0.05 when untreated and treated ECs are compared. There is not statistical difference when treated and untreated macrophages are compared. (C) TGF-β measurement in the culture supernatants of *M. avium*-infected cells. (Hatched bars) SB431542-treated macrophages, (grey bars) SB431542-treated ECs, untreated macrophages (black bars), untreated ECs (open bars). SB431542 (100 µM) was added 1 h before cell/bacilli co-incubation. *P<0.05 when treated macrophages were compared to untreated ones and ^#^ *P*<0.05 when treated ECs were compared to untreated ones at each time point. Results from one experiment among two carried out in duplicates.

The ability of ECs to overproduce TGF-β even during the course of *M. avium* infection suggests that high levels of this cytokine play a role inhibiting *bacilli* replication in infected-cells in an autocrine fashion. To test this hypothesis, we first checked whether treatment of infected-undifferentiated macrophages with higher concentration of TGF-β could overcome *M. avium* growth like we observe in cultures of infected-ECs. Therefore, macrophages were treated with 100 pg/ml of recombinant TGF-β (rTGF-β) 4 h post-infection and then the same concentration of rTGF-β was added each 24 h during the course of infection. The concentration of rTGF-β used in this experiment was based on the concentration of TGF-β secreted in the conditioned medium (∼150 pg/ml) from ECs cultures, which is higher than the TGF-β concentration found in the conditioned medium from undifferentiated macrophages cultures (∼50 pg/ml) [25]. As shown in Fig. 3A, treatment of macrophages with 100 pg/ml of rTGF-β overrode macrophages capability to increase *M. avium* replication when compared to untreated cells. In addition, to rule out that rTGF-β itself affects *bacilli* replication, we performed a growth curve of *M. avium* in the presence or absence of different concentrations of rTGF-β. Our result showed that even in the presence of high concentrations of rTGF-β, *M. avium* growth was not modulated by the presence of the cytokine (Fig. S1).

To verify the functional role of TGF-β signaling itself, macrophages and ECs were treated with a potent and selective inhibitor of TGF-β type I receptor kinase activity [28]. SB-431542 is a very stable inhibitor being effective for up to 60 h in inhibition assays [28]. Therefore, macrophages and ECs were pre-treated with SB-431542 for 1 h and then infected with *M. avium*. As shown in Fig. 3B, treatment with SB-431542 significantly increased the intracellular growth of *M. avium* in infected-ECs, suggesting involvement of TGF-β signaling in the regulation of intracellular *M. avium* replication. Although in this experiment infected macrophages treated with SB-431542 showed a slight decrease in *bacilli* growth (Fig. 3B), this effect was not statistically significant when compared to untreated macrophages. Considering that infected-macrophages produce much less TGF-β than infected-ECs, it is possible that the amounts of TGF-β secreted by these cells may not be sufficient to stimulate TGF-β signaling to an extent that is necessary for the inhibition of *M. avium* replication. Therefore, even after inhibition of receptor activity with SB-431542, it was expected that the rates of *M. avium* growth would remain somewhat unchanged in infected-macrophages. Additionally, we also quantified TGF-β secretion in the culture medium from cells submitted to the experiment described above. As expected, TGF-β levels increased during the kinetics of infection in untreated-ECs cultures, whereas in untreated-macrophages the levels were much lower (Fig. 3C). In cultures of infected-ECs treated with SB-431542, TGF-β levels were drastically diminished compared to untreated infected-ECs. In SB-431542-treated infected macrophages TGF-β levels were not altered compared with their untreated counterparts, except at 4 h post-infection were TGF-β levels significantly dropped (Fig. 3C). Together, these results suggest that signaling through TGF-β receptor is not only involved in the regulation of intracellular *M. avium* growth, but also points to the presence of an autoregulatory feedback loop of TGF-β production, since inhibition of TGF-β receptor activity correlated with diminished TGF-β production in ECs.

### TGF-β-mediated inhibition of *M. avium* growth involves activation of ERK1/2

**Figure 4:**
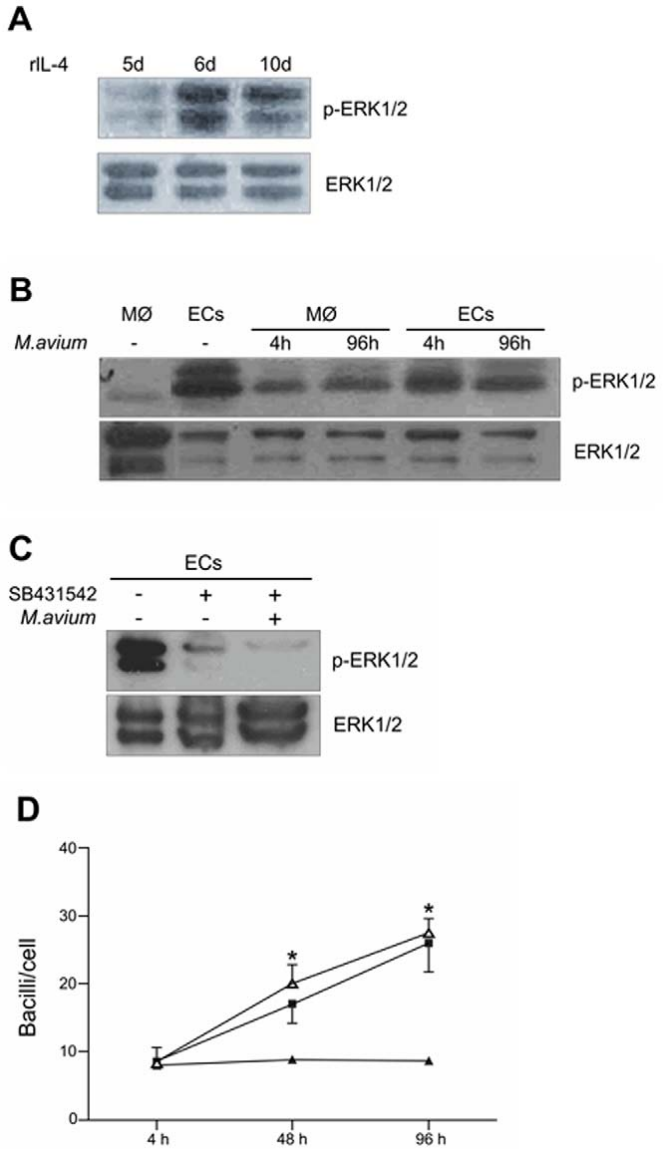
ERK1/2 is involved in TGF-β-mediated inhibition of *M. avium* replication. (A) Macrophages were pulsed with rIL-4 on day one and after 5, 6 and 10 days, cells were collected and submitted to immunoblotting for detection of ERK1/2 phosphorylation. Total ERK1/2 was detected as control for protein loading. (B) Macrophages and ECs were infected or not with *M. avium* at the indicated times post-infection and phosphorylation of ERK1/2 was detected by immunoblotting. Total ERK1/2 was detected as control for protein loading. (C) ECs were either infected or not with *M. avium* and pre-treated or not with SB431542 one hour before infection. Four hours post-infection cells were collected and submitted to immunoblotting for detection of ERK1/2 phosphorylation. Total ERK1/2 was detected as control for protein loading. (D) Kinetics of *M. avium*-infection on ECs (full triangles), macrophages (full squares) and ECs treated with U0126 (10 µM) 24 h before infection (open triangles). *P≤0.05 when macrophages and U0126-treated ECs are compared with untreated ECs.

To check whether TGF-β-mediated inhibition of *M. avium* growth in infected- ECs involves the activation of ERK1/2 MAPK, we first analyzed the status of ERK1/2 phosphorylation during the course of differentiation of macrophages into ECs. Therefore, macrophages were pulsed with rIL-4 on day 1 and after 5, 6 and 10 days cells were harvested for Western blot analysis of ERK1/2 phosphorylation. As shown in Fig. 4A, a strong phosphorylation of ERK1/2 was observed on day 6, which remained highly phosphorylated on day 10, indicating that ERK1/2 phosphorylation is induced at the final steps of the differentiation process, remaining highly phosphorylated after differentiation.

We also investigated the status of ERK1/2 phosphorylation in infected-cells. Macrophages and ECs were either infected or not with *M. avium* and 4 h and 96 h post-infection cells were harvested for analysis of ERK1/2 phosphorylation. Figure 4B shows that in contrast to uninfected-macrophages, ERK1/2 was highly phosphorylated in uninfected-ECs. Phosphorylation of ERK1/2 was induced in macrophages 4 h post-infection, which slightly increased 96 h post-infection. On the other hand, infected-ECs showed similar band intensities of ERK1/2 phosphorylation in both times post-infection, which were as intense as in uninfected-ECs, however stronger than in infected-macrophages. This result suggests that ECs achieved a saturation of ERK1/2 activation that was no longer increased upon infection with *M. avium*, a condition that was opposed in macrophages where ERK1/2 activation was induced only after infection. These different modulations of ERK1/2 activity between ECs and macrophages imply that a proper balance in the magnitude and duration of ERK1/2 activity might be determinant in providing a specific intracellular *milieu* for the control of *M. avium* replication.

To check whether ERK1/2 activity in ECs was dependent on the activation of the TGF-β receptor signaling, ECs were pre-treated with the TGF-β receptor inhibitor SB-431542 and submitted to infection with *M. avium* for evaluation of ERK1/2 phosphorylation. As shown in Fig. 4C, ERK1/2 phosphorylation was greatly inhibited by SB-431542, irrespective if ECs were infected or not. Moreover, to test if ERK1/2 activation was playing a role in the inhibition of *M. avium* growth, ECs were pre-treated with U0126, a well-established inhibitor of the ERK1/2 pathway, and then infected or not with *M. avium*. Figure 4D shows that replication of *M. avium* was significantly increased in U0126-treated ECs, an effect that was similar to the replication rates seen in untreated infected-macrophages, suggesting that enhanced ERK1/2 activity indeed favors the inhibition of *M. avium* growth in ECs.

### Inhibition of intracellular pathogens growth in ECs is pathogen-dependent

**Figure 5:**
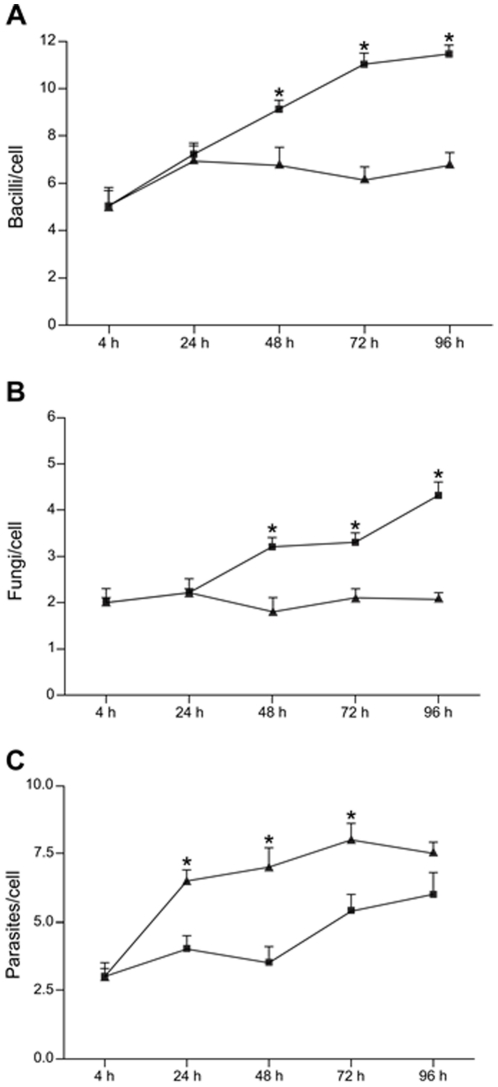
*M. avium* and *P. brasiliensis* but not *L. amazonensis* replication is inhibited in infected-ECs. Kinetics of infection in macrophages (squares) and in ECs (triangles) expressed as pathogen/cell. (A) *M. avium* (rate of infection 50∶1). (B) *P. brasiliensis* (rate of infection 5∶1). (C) *L. amazonensis* (rate of infection 2∶1). Results are representative of 3 experiments done in duplicates. *P<0.05 when macrophages and ECs are compared at each time point.

**Figure 6:**
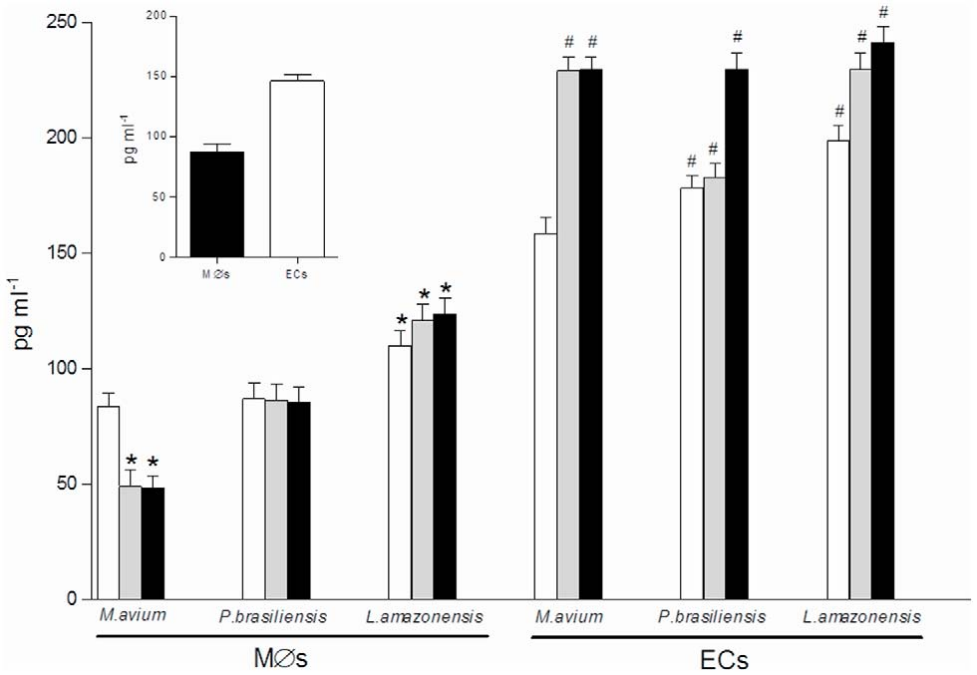
Infected-ECs secrete high levels of TGF-β irrespective of the nature of the infecting pathogen. TGF-β measurement in the culture supernatants of macrophages and ECs infected with different pathogens, as indicated, at 3 time points after infection: open bars, 4 h; grey bars, 48 h and black bars, 96 h. Insert: basal levels of TGF-β in macrophages (black bar) and ECs (open bar) in the absence of infection. Results are representative of two experiments done in duplicates. *P<0.05 when compared to uninfected macrophages and ^#^ *P*<0.05 when compared to uninfected ECs.

To gain further insight into the mechanism involved in the control of *M. avium* replication in ECs, we questioned whether this effect was pathogen-dependent. To answer this question we choose two taxonomically unrelated intracellular pathogens, including the dimorphic fungus *Paracoccidioides brasiliensis* (*P. brasiliensis*) and the protozoan parasite *Leishmania amazonensis* (*L. amazonensis*). Both pathogens are known to infect mononuclear phagocytes and also to induce granuloma formation in infected mice [29], [30], [31]. Therefore, macrophages and ECs were submitted to infection either with *P. brasiliensis*, *L. amazonensis* amastigotes or *M. avium* to assess the intracellular growth behavior of these pathogens in infected cells. As shown in Fig. 5, four hours post-infection, the uptake of each pathogen into macrophages and ECs was similar. The kinetics of infection revealed that like *M. avium*, replication of *P. brasiliensis* was counteracted in ECs (Fig. 5 A and B). In contrast, the number of *L. amazonensis* parasites in ECs underwent replication above the rates seen in infected-macrophages (Fig. 5 C). Additionally, we quantified TGF-β secretion in the culture medium from cells submitted to infection with the pathogens described above. As shown in Fig. 6, like *M. avium*, both *P. brasiliensis* and *L. amazonensis* induced increased TGF-β secretion in infected-ECs cultures, showing similar levels of the cytokine 96 h post-infection. Although TGF-β levels from cultures of *L. amazonensis*-infected macrophages were significantly higher compared to macrophages infected either with *M. avium* or *P. brasiliensis*, the overall production of TGF-β in infected-macrophages were still lower compared to infected-ECs. Taking together, these results show that in ECs TGF-β-mediated inhibition of intracellular parasites growth is pathogen-dependent.

## Discussion

The persistence of *M. avium* at sites of infection is due in part to high resistance of these microorganisms to microbicidal devices of host macrophages. Once within the macrophage, this facultative intracellular pathogen must commandeer the host cell machinery to enable its own multiplication and survival. However, the mechanisms by which this is accomplished are largely unknown. One possible device implicated in this effect might involve immunoregulatory mediators such as TGF-β, since it has been shown that this cytokine is endogenously produced by macrophages infected with *M. avium* [32], [33], [34], [35]. It is believed that TGF-β favors intracellular *M. avium* growth by impairing the response of macrophages to cytokines such as TNF-α or by suppression of nitric oxide (NO), and reactive oxygen intermediates [12], [13], [14]. The data presented in this report have shown that the role of TGF-β favoring intracellular microbial growth is not that straightforward, but greatly depends on the amplitude of TGF-β signaling coordinating the strength and duration of ERK1/2 activity.

We used epithelioid cell surrogates (ECs) to investigate the biological role of TGF-β interfering with the intracellular growth of *M. avium*, since these cells produce high levels of TGF-β and are efficient in inhibiting the intracellular growth of *M. avium* [25]. ECs were established from primary murine peritoneal macrophages through a process of differentiation induced by recombinant IL-4 (rIL-4) and are distinct from dendritic cells differentiated from human peripheral blood monocytes using rIL-4/rGM-CSF [24], [25]. In this report, we add new insight about the functional receptor involved in the signaling pathway leading to macrophage differentiation into ECs. We have demonstrated that like rIL-4, treatment with rIL-13 also induced morphological and functional differentiation of macrophages into ECs. In fact, both IL-4 and IL-13 receptors are multimeric and share at least one common chain called IL-4Rα. The IL-4Rα chain binds IL-4 leading to dimerization with proteins to form either a type I or type II receptor. The type I receptor arises by recruitment of a common gamma chain (γC) while the type II is formed by interaction of IL-4Rα with IL-13Rα1 instead of γC [26], [27]. IL-13 binds to IL-13Rα1 inducing heterodimerization with IL-4Rα to form a complex identical to the type II IL-4R, which is able to transduce both IL-4 and IL-13 signals [36]. Therefore, our results suggest that the type II IL-4R is the functional receptor involved in the signaling pathway leading to macrophage differentiation into ECs, thus explaining the striking overlapping actions of IL-4 and IL-13 in our model.

To demonstrate that high TGF-β levels produced by ECs play a role in the inhibition of intracellular *M. avium* growth, we used two strategies. First, we treated infected-macrophages with a concentration of rTGF-β that is related to the concentration of TGF-β secreted in the culture medium of ECs culture. Our results showed that increased TGF-β levels overrode macrophages capability to enhance intracellular *M. avium* growth, similarly to that observed in infected-ECs. Second, we blocked TGF-β receptor kinase activity in infected-ECs with SB-431542, which significantly increased bacterial replication rates. Interestingly, in contrast to SB-431542-treated infected macrophages, TGF-β levels were drastically diminished in the culture medium of infected-ECs treated with SB-431542, suggesting that signaling through TGF-β receptor is not only involved in the inhibition of *M. avium* growth, but also that high TGF-β levels act autocrinally to regulate its own expression in ECs. This positive autocrine loop of TGF-β may be necessary to stimulate TGF-β signaling to an extent that favors the inhibition of *M. avium* replication, suggesting that the amplitude of TGF-β signaling is an important determinant of infection outcome.

TGF-β exerts its effects through heteromeric receptor complex consisting of type I and type II transmembrane serine/threonine kinase receptors. Smad family members and MAPKs such as ERK1/2 have been implicated in the signaling by TGF-β [37], [38], [39]. Moreover, there is an emerging consensus that replication of *M. avium* in murine macrophages is inhibited by activated ERK1/2 [21], [40]. Our data demonstrated that during the course of differentiation of macrophages into ECs ERK1/2 phosphorylation was induced on day 6, remaining phosphorylated after completion of differentiation, showing that unlike macrophages ERK1/2 activity is sustained in ECs. We do not know yet the mechanism responsible for this effect. However, based on our data showing a time-dependent increase of TGF-β production during the course of macrophages differentiation (Fig. 2B), we argue that the amounts of TGF-β produced from day 6 on could be the critical threshold concentration of TGF-β necessary to stimulate TGF-β signaling to an extent that is capable to induce and maintain long-term ERK1/2 activity in ECs. This magnitude of ERK1/2 activation could in turn modulate ECs function towards the inhibition of *M. avium* growth. Indeed, when we checked ERK1/2 phosphorylation in *M. avium* infected-cells, we showed that in ECs high levels of phosphorylated ERK1/2 remained almost unchanged even upon infection, a condition that was opposed in macrophages in which ERK1/2 phosphorylation was induced only after infection. Interestingly, blocking TGF-β receptor activity with SB-431542 greatly inhibited ERK1/2 phosphorylation in infected as well as in uninfected-ECs, showing that ERK1/2 activity is dependent on TGF-β signaling. Moreover, inhibition of ERK1/2 activity with U0126 significantly increased *M. avium* replication rates in infected-ECs. Together, these results suggest that a proper balance in the magnitude and duration of ERK1/2 activity may be determinant for infected-cells to control mycobacterial growth.

In effect, there is an emerging concept from the recent literature placing ERK1/2 as an important regulator of *M. avium* replication in macrophages. It has been shown that inhibition of the ERK1/2 but not of the p38 pathway further enhanced intracellular growth of highly replicative *M. avium* strain, showing that pathogenic mycobacteria have evolved mechanisms to prevent a sustained activation of ERK1/2, and this may account for their intracellular growth [21]. Despite recent success there are still significant gaps in the literature whether replication of *M. avium* is inhibited directly by ERK1/2 or indirectly by inflammatory products of ERK1/2 activation. For example, inhibitors of ERK1/2 such as PD98059 inhibits p47 (phox) phosphorylation, an important enzyme involved in the formation of reactive oxygen species important for controlling bacterial replication [41]. Also PD98059 and U0126 lead to decreased secretion of cytokines such as TNF-α thus further enhancing the growth of pathogenic mycobacteria in human macrophages [21]. Considering our results showing that ERK1/2 is highly active in *M. avium*-infected ECs compared to infected-macrophages, one could speculate that a direct correlation between high ERK1/2 activity and high levels of TNF-α could exist, therefore placing TNF-α as a mediator of ERK1/2 in the inhibition of *M. avium* growth in ECs. However, as previously showed by our group the cytokine secretion profile in undifferentiated macrophage and ECs revealed that IFN-γ was almost undetected in the supernatants of both cultures, irrespective if cells were infected or not with *M. avium* [25]. Also, undifferentiated macrophages and ECs similarly produced low TNF-α levels which were increased in a time dependent manner in infected-macrophages but not in infected-ECs [25]. Therefore, although macrophages and ECs diverge in relation to TGF-β production, IFN-γ and TNF-α secretion levels were low in both cell types, suggesting that endogenous TGF-β is not involved in the regulation of these cytokines. Hence, the role of IFN-γ and TNF-α controlling *bacilli* replication may not be detrimental in our cell system [25]. In this context, a recent report by Klug *et al.* [40] has shown that replication of *M. avium* in macrophages is directly influenced by ERK1/2 activity independently of TNF-α or IL-10, two products of MAPK activation. In this study, the authors showed that exogenous addition of TNF-α to TNF-α-deficient *M. avium*-infected macrophages did not override the replication-promoting effect of ERK1/2 inhibitor PD98059. Therefore, our results showing that macrophages and ECs differ in the magnitude and duration of ERK1/2 activity not only support an important role for ERK1/2 in the control of *M. avium* replication, but also add new insight about a growing concept that a qualitative and quantitative ERK1/2 activity may generate variations in signaling output that regulate cell fate decisions and therefore distinct biological outcomes [42].

In addition to *M. avium*, the interaction of other granuloma-inducing pathogens (*P. brasiliensis* and *L. amazonensis*) with ECs showed that inhibition of intracellular parasites growth was pathogen-dependent. Like *M. avium*, replication of *P. brasiliensis* was counteracted in ECs, while *L. amazonensis* parasites underwent replication above the rates seen in infected-macrophages. We showed that TGF-β production by infected-ECs was similarly high, irrespective of the nature of the infective pathogen, suggesting that TGF-β-mediated activation of ERK1/2 might exist in all infected-ECs. Therefore, it is possible that while high levels of activated ERK1/2 contribute to the control of intracellular replication of *M. avium* and *P. brasiliensis*, they may not be sufficient to override *L. amazonensis* amastigotes growth. However, replication of *L. amazonensis* amastigotes were enhanced rather than inhibited in infected-ECs. We are currently investigating the mechanism involved in this effect. We hypothesize that an inhibitory effect induced by *L. amazonensis* itself in the control of ERK1/2 activity may contribute to this effect. In line with this argument, several studies have indicated that limited ERK phosphorylation correlates with infection as both *L. amazonensis* amastigotes and *Leishmania donovani* promastigotes have been shown to prevent ERK1/2 activation while *Leishmania mexicana* has been shown to promote ERK1/2 degradation [43], [44], [45].

**Figure 7:**
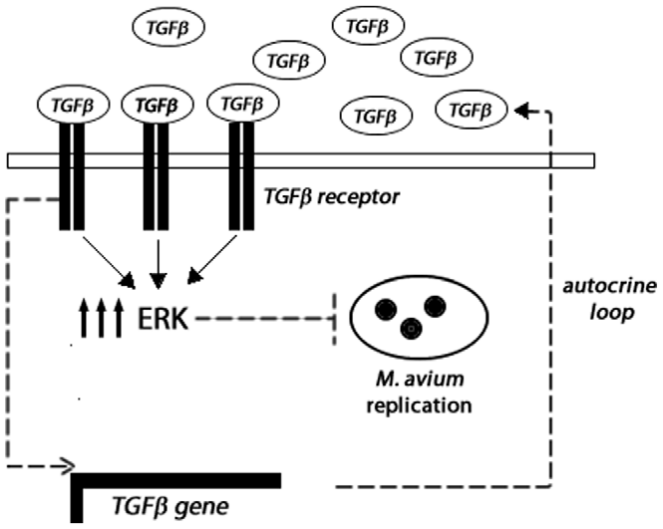
Schematic model of TGF-β-mediated inhibition of *M. avium* growth in infected-ECs. ECs produce high levels of TGF-β, which in turn induce TGF-β receptor signaling leading to increased ERK activity and also to auto-induction of TGF-β gene by an unknown mechanism, resulting in an autocrine loop of activation. Increased and sustained ERK1/2 activity impairs *M. avium* replication.

A variety of clinical and experimental studies in many infectious disease systems have demonstrated potent effects of TGF-β in the regulation of microbial replication and host responses to pathogens. In general, most pathogens that infect macrophages including *Toxoplasma gondii*, *Leishmania amazonensis*, *Trypanosoma cruzi* and the *M. avium* complex have evolved mechanisms to induce TGF-β production, and TGF-β production in turn suppresses the killing activity of macrophages, enhances intracellular proliferation of the pathogen, and thus favors virulence [46], [47], [48]. Most of the effects of TGF-β in promoting intracellular proliferation of pathogens in infected-macrophages have been associated with its indirect immunosuppressive properties [12], [13], [14]. Although our results with infected-undifferentiated peritoneal macrophages showed a tendency for diminution of TGF-β levels (Figures 2C, 3C and 6) and this was followed by increased *M. avium* replication, it is important to consider that TGF-β production by infected-macrophages may vary depending on the macrophages sources, on distinct strains of *M. avium* as well as on the time course of infection employed for detection of TGF-β production [49], [50]. Moreover, some studies have demonstrated that TGF-β endogenously produced by monocytes or macrophages were ineffective in modulating anti-*M. avium* activity in infected-cells [51], [52], [53]. Therefore, our data showing that the magnitude and duration of ERK1/2 activity are determinant to control intracellular *M. avium* replication bring new perspectives to understand the influence of TGF-β on host response depending on the context of ERK1/2 regulation. We showed that enhanced production of TGF-β by ECs regulates high and sustained levels of ERK1/2 activity which favors the inhibition of *M. avium* replication. We also propose that an autocrine positive loop for TGF-β in the control of ERK1/2 activity might exist, since impairment of TGF-β signaling not only downregulated TGF-β production but also ERK1/2 activity in ECs (Fig. 7). Although the implication of this autocrine loop seems to be of singular importance in the inhibition of *M. avium* growth in ECs *in vitro*, further studies are required to evaluate its contribution in granulomas *in vivo*. The granuloma structure is maintained and stabilized by events mediated by both host and pathogen. It is beneficial for the host as it helps to contain the infection to localized regions. It is believed that bacteria can live for prolonged periods of time within the environment of a granuloma [11], while at the same time bacterial spreading to other areas of the organism is restricted [54]. The mature granuloma is typically an area of localized infection characterized by high levels of immunological activity [55]. TGF-β has been found in epithelioid cells and in giant cells during the morphogenesis of granulomas [56]. Therefore, an appropriate TGF-β signaling may be one strategy to keep intracellular pathogens replication under control and might also contribute to maintain the infection in equilibrium during the formation of granulomas harboring particular pathogens.

## Materials and Methods

### Animals

BALB/c pathogen free mice were obtained from the Center for the Development of Experimental Models (CEDEME, UNIFESP, São Paulo, SP, Brazil). All mouse procedures performed in this study were reviewed and approved by the Institutional Ethics Committee for Experimental Research of Federal University of Sao Paulo (protocol # 01685/05).

### Isolation and cultivation of resident peritoneal macrophages and generation of ECs

Resident macrophages from 12-week-old BALB/c male mice were obtained by rinsing the peritoneal cavity with 5 ml of cold Hank's Balanced Salt Solution ( HBSS) (Cultilab, Campinas, SP, Brazil). Macrophages were stained with crystal violet and counted in a Neubauer chamber. Cells were ressuspended in RPMI-1640 medium (Cultilab) and unless otherwise stated distributed on glass coverslips placed into 24-well plates (5×10^5^ cells/well). For all experiments, the suspension of peritoneal cells was left to adhere for 30 min at 37°C in 5% CO_2_ atmosphere and then washed thrice with PBS to remove nonadherent cells. The adhered cell population was cultivated in complete RPMI medium buffered with 10 mM HEPES plus 24 mM sodium bicarbonate, and supplemented with 5% calf fetal serum (Cultilab), 40 mg l^−1^ gentamicin and in the presence or not of 50 ng ml^−1^ rIL-4 or 10 ng ml^−1^ rIL-13 (PeproTech Inc., Rocky Hill, NJ, USA) for 7 days, as described [24].

### *Mycobacterium avium* culture

A virulent smooth transparent strain of *M. avium*, 62TL, was grown in Middlebrook 7H9 liquid medium (Difco, Detroit, MI, USA) supplemented with 10% OADC (oleic acid, albumin, dextrose and catalase) (BD, Franklin Lakes, NJ, USA) for 15–20 days [57]. A standard curve relating the O.D._560_ of the liquid culture suspension and the number of corresponding colony forming units (CFU) per ml, obtained through plating in 7H10 (Difco) solid medium (plus 10% OADC), was established to estimate bacterial concentration. Aliquots of liquid medium log-phase cultures were kept frozen without cryoprotector at −70°C until use. The viability of the bacilli maintained as frozen stocks was evaluated month by month during a year. For the experiments, bacilli frozen up for two months, the most, were employed. The frozen stocks were always thawed and immediately ressupended in RPMI medium and the concentration of bacilli was adjusted accordingly to a viability curve which was established from frozen aliquots of *M. avium* thawed month by month. Cell suspensions were serially diluted and plated on 7H10 solid medium enriched with 10% OADC to count CFUs when colonies were observed with naked eyes.

### In vitro infection and analysis of mycobacterial growth

Seven days cultures of macrophages and ECs on glass coverslips were washed three times with PBS to remove remaining culture medium. A suspension of *M. avium* 62TL strain, in gentamicin/FCS-free RPMI medium, was added at a ratio of 50 bacilli/cell to the culture plates [58]. After 4 h of co-incubation, supernatants were collected and non-internalized bacteria were eliminated by washing three times with PBS. Cultures were replenished with complete RPMI medium and followed for up to 96 h. Both, supernatants and glass coverslips, were harvested at different times. Supernatants were kept at −70°C until use and the cells over glass slides were fixed with pure methanol and Ziehl Neelsen stained. The number of bacteria inside both cell types and the percentage of infected cells were calculated by observation under a photomicroscope (Axiolab, Carl Zeiss, Germany).

### Measurement of TGF-β

TGF-β levels present in the culture supernatants of macrophages and ECs were quantified by a sandwich ELISA kit (TGF-β1 Duo Set® kit) following the manufacturer's instructions (R&D Systems, Minneapolis, MN, USA) and analysed at 492 nm (ELISA reader; LabSystems Multiskan MS, Markham, Ontario, Canada).

### TGF-β treatment of macrophages followed by *M. avium* infection

Peritoneal macrophages were maintained for 7 days in culture and then rTGF-β (PeproTech Inc.) was added or not to the cultures, at a final concentration of 100 pg ml^−1^ 4 h post-infection with *M. avium*. Each 24 h post-infection cells were washed twice with PBS and cultures were then replenished with complete RPMI medium in the presence of 100 pg ml^−1^ rTGF-β during the course of infection. Cells were always maintained at 37°C and 5% CO_2_ atmosphere. Culture supernatants and glass coverslips were harvested at each time point either for measurement of TGF-β secretion or for mycobacterial growth, as described above.

### Inhibition of TGF-β receptor kinase activity

Macrophages and ECs were serum starved overnight and then pre-incubated or not with the TGF-β receptor kinase inhibitor, SB431542 (Sigma, St. Louis, MO, USA), at a final concentration of 10 µM, 1 h before *M. avium* infection. After that period cells were washed twice with PBS and then submitted or not to infection with *M. avium* in completed medium. Culture supernatants and glass coverslips were harvested at each time point during the kinetics of infection either for measurement of TGF-β secretion or for mycobacterial growth, as described above. For detection of ERK1/2 phosphorylation in ECs treated with SB431542, ECs were serum starved overnight and then pre-treated or not with 10 µM SB431542 for 1 h. After that period cells were washed twice with PBS and then submitted or not to infection with *M. avium* in medium without serum. Four hours post-infection cells were harvested and total cell extract were produced for evaluation of ERK1/2 phosphorylation by immunoblot.

### Immunoblot analysis of ERK ½ phosphorylation

For these assays 3×10^7^ cells were seeded in 100×20 mm Petri dishes. Total proteins from macrophages, infected or not with *M. avium* (50∶1) and treated or not with cytokines or pharmacological inhibitors were extracted by addition of 100 µl lysis buffer (10% NP-40, 150 mM NaCl, 50 mM Tris, 500 mM NaF) pH 7.4. Protease inhibitors (1 mM PMSF, 10 µg ml^−1^ leupeptin and 10 µg ml^−1^ aprotinin) were always added to the lysis buffer immediately before use. Protein extracts were obtained from cells treated or not with rIL-4, or with the TGF-β receptor kinase inhibitor SB431542. Samples were loaded under reducing conditions on 10% SDS-polyacrylamide gels and then transferred to PVDF membranes. After blockage with 5% defatted milk in TRIS-buffered saline containing 0.1% Tween 20 (TTBS) (Sigma) for 1 h, membranes were incubated with murine monoclonal antibodies against phospho-ERK1/2 (Santa Cruz Biotechnology, Santa Cruz, CA, USA) or rabbit polyclonal antibodies against ERK1/2 (Cell Signaling Technology, Danvers, MA, USA) overnight at 4°C. Membranes were washed thrice with TTBS and incubated for 1 h with the required horseradish peroxidase-conjugated antibodies (Sigma). The activity of membrane-bound peroxidase was detected by using enhanced chemiluminescent method (Pierce Biotechnology, Rockford, IL, USA).

### Inhibition of ERK1/2 phosphorylation with U0126

ECs were treated or not with 10 µM U0126 (Calbiochem, La Jolla, CA, USA) for 24 h in medium without serum before *M. avium* infection. After that period cells were washed twice with PBS and then submitted or not to infection with *M. avium* in completed medium. At each time point during a kinetic of 96 h of infection cells were harvested and mycobacterial growth was evaluated as described above.

### Infection of macrophages and ECs cultures with other pathogens

Macrophages and ECs, cultivated on glass coverslips placed into 24-well plates, were incubated separately with a highly virulent isolate of *Paracoccidioides brasiliensis* (Pb isolate 18), at a ratio of 5 fungi/cell [59] and *Leishmania amazonensis*, at a ratio of 2 amastigotes/cell [60]. The following steps were identical to those mentioned above for *M. avium*. Material was fixed with methanol for *L. amazonensis* infected cells and with 0,25% glutaraldehyde (Sigma) for *P. brasiliensis*. Giemsa staining was employed for *L. amazonensis* infected cells and none to analyse cells infection with *P. brasiliensis*. The number of *L. amazonensis* inside infected cells was counted in a Zeiss photomicroscope and *P. brasiliensis* in an Olympus phase contrast microscope (CBA213 model).

### Statistical analysis

The unpaired t-test was used to analyze data obtained from experiments where two cell culture conditions were compared. For all analysis, *P*<0.05 was considered statistically significant.

## Supporting Information

Figure S1 ***M. avium*** **62TL strain growth curve in the presence of different concentrations of rTGF-β.** *M. avium* were inoculated in 7H9-10% OADC medium in the presence or absence of different concentrations of rTGF-β. Untreated (full circle), 10 ng/ml rTGF-β (full square), 5 ng/ml rTGF-β (full triangle), 2,5 ng/ml rTGF-β (open triangle). Optical density of each culture was measured at 560 nm. Mean values are plotted against time (in days).
(TIF)
