# Supplementary figures and images for: TGF-β-Mediated Sustained ERK1/2 Activity Promotes the Inhibition of Intracellular Growth of Mycobacterium avium in Epithelioid Cells Surrogates
Source: PLoS One. 2011 Jun 22;6(6):e21465. doi: 10.1371/journal.pone.0021465 (PMC3120888; doi:10.1371/journal.pone.0021465)

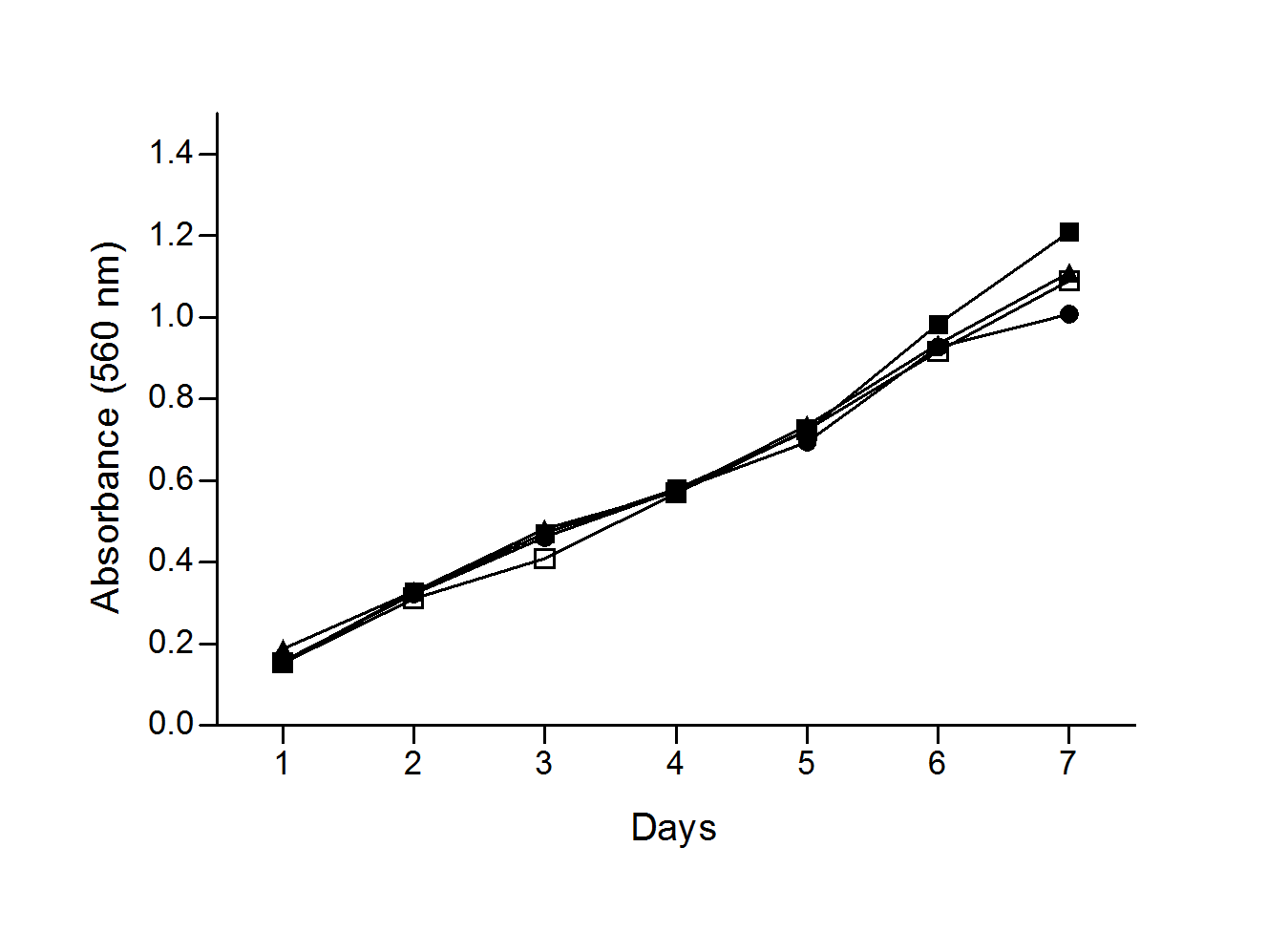

Supplement: Figure S1 — M. avium 62TL strain growth curve in the presence of different concentrations of rTGF-β. M. avium were inoculated in 7H9-10% OADC medium in the presence or absence of different concentrations of rTGF-β. Untreated (full circle), 10 ng/ml rTGF-β (full square), 5 ng/ml rTGF-β (full triangle), 2,5 ng/ml rTGF-β (open triangle). Optical density of each culture was measured at 560 nm. Mean values are plotted against time (in days). (TIF) [file pone.0021465.s001.tif]
